# Inclusive groups can avoid the tragedy of the commons

**DOI:** 10.1038/s41598-020-79731-y

**Published:** 2020-12-28

**Authors:** Arend Hintze, Jochen Staudacher, Katja Gelhar, Alexander Pothmann, Juliana Rasch, Daniel Wildegger

**Affiliations:** 1 Institute for Complex Dynamical Systems and MicroData Analytics, Dalarna University, Falun, Sweden; 2 BEACON Center for the Study of Evolution in Action, Michigan State University, East Lansing, USA; 3 Faculty of Computer Science, Kempten University of Applied Sciences, Kempten, Germany

**Keywords:** Evolution, Computational models

## Abstract

The public goods game is a famous example illustrating the tragedy of the commons (Hardin in Science 162:1243–1248, 1968). In this game cooperating individuals contribute to a pool, which in turn is distributed to all members of the group, including defectors who reap the same rewards as cooperators without having made a contribution before. The question is now, how to incentivize group members to all cooperate as it maximizes the common good. While costly punishment (Helbing et al. in New J Phys 12:083005, 2010) presents one such method, the cost of punishment still reduces the common good. The selfishness of the group members favors defectors. Here we show that including other members of the groups and sharing rewards with them can be another incentive for cooperation, avoiding the cost required for punishment. Further, we show how punishment and this form of inclusiveness interact. This work suggests that a redistribution similar to a basic income that is coupled to the economic success of the entire group could overcome the tragedy of the commons.

## Introduction

The *tragedy of the commons*^1^ is a well studied model in which the interests of the group are pitched against the interests of the individuals. Individuals either chose to contribute to the common good (cooperate) with a single payment, or withhold their investment (defect) of said investment. The common good can experience a growth in value due to synergy, which consequently benefits everyone, also the defectors. In the end, tragically, defectors will always receive a higher reward than the cooperators, even though a higher total gain could be achieved if everyone would cooperate in the first place.

As such, this model has been extensively studied to describe social systems, in which for example taxes represent the common good, and tax evaders would be defectors of that game. Obviously, we are interested in methods which encourage everyone to cooperate, overcoming the individual benefit gained from defecting. Many different solutions have been identified which promote cooperation, such as reciprocity^3^, green beard effects^4^, or costly punishment of defectors^5–7^. Similarly, we know that in games played spatially cooperation often dominates^2^ compared to well mixed situations. Besides spatial play, the easiest method to incentivize cooperation in humans^8^ seems to be punishment (for a more detailed description of this rather wide term see Raihani and Bshary^7^). Punishment, which in its ability to drive cooperation by direct or indirect reciprocity^9^, can take many forms^10^ and differs between humans and other organisms^11^. Here we will consider costly punishment, which not only imposes a cost on the defector, but also requires the punishing agent to come up for the cost of punishment. As this form of punishment is an established form of driving cooperation it serves as the basis for further comparisons.

Individuals engaging in the public goods game selfishly optimize their own rewards, while neglecting the common good. One concept, also derived from nature, that might be able to overcome this issue is group-level selection, where the payoff of the individual is not only dependent on its own choices, but also of that of the group. Evolution is normally selecting the individuals of a population according how well fit they are to their environment, as they produce the most viable offspring. However, organisms often form groups to take advantage of mutual benefits that such behavior grants. Fighting off enemies by swarming, division of labor, or other forms of collaboration come to mind. If not only individuals experience the benefit, but the group as a whole enjoys reproductive success over another group, we speak of group-level selection.

The slime mold *Dictyostelium discoideum*^12^ and its life cycle illustrates the difference between individual and group-level selection. In its amoeba stage, cells can replicate individually, and evolution occurs on the level of the individual. When food becomes sparse, cells aggregate and first form a mobile slug which later culminates into a fruiting body. The group of cells forming the fruiting body can now experience the rewards of group-level selection when wind disperses the spoors. The individual spores in turn become amoebas again, and so forth. Group-level selection in the strictest sense requires all members to be selected and being allowed to propagate offspring into the next generation.

When organisms receive benefits from hunting together, for example hyenas^13^, the situation becomes more complicated. The group receives a benefit driving cooperation^14^, however, the group does not strictly reproduce as a whole. Mechanisms like kin selection, multilevel selection, and inclusive fitness come into play^15^. While these are distinct concepts, they are often used interchangeably. Kin selection would require the members of a group to also be selected by their genetic distance, which we do not consider here. Inclusive fitness on the other hand refers to a much larger concept. In predator prey dynamics the fitness of the prey is dependent on the fitness of the predator leading to the “fit when rare” phenomena for example. Meanwhile, multilevel selection refers to situations where selection occurs on a much higher level than the individual, which has been identified as one reason for the evolution of multicellularity^16^. Regardless, all these mechanisms in one way or the other affect cooperation^3^ but remain highly debated concepts^17^.

Here specifically, we are interested in how humans might be able to overcome the tragedy of the commons. Even when working in groups, and payoffs are dependent on synergistic behavior of the group, individuals still reproduce individually, precluding a group-level mechanism from taking effect. However, resources within the group can be redistributed. Instead of assuming individuals to be selfish, we can assume or force the group members to be inclusive, and thus have group members share rewards with each other. A fully inclusive group would pool all rewards individually obtained, and then shares them equally amongst its members. A group of only selfish members would not pool their rewards. It is easy to imagine a mixed model between those extremes^18^. The degree of selfishness, or its opposite inclusiveness, can define how much of the rewards are distributed equally. The pooling of resources to allow for group-level selection in the public goods game has been introduced earlier^19^ but not its fractional redistribution. Further, groups here still do not reproduce as a group but remain individual replicators. The fraction of payoffs that can be pooled or remain at the individual can be dialed. We call this fraction the degree of selfishness $\zeta$, and consequently inclusiveness becomes $1.0-\zeta$. Imagine the degree of selfishness to be $\zeta =0.5$. In that case, 50% of the payoff each individual receives would be pooled and redistributed equally, while the other 50% of the payoff remains with the individual without being redistributed. In the extreme case of $\zeta =0.0$ all payoff (the net earnings of the group) is redistributed effectively making it a fully inclusive group. The expectation is that different degrees of inclusiveness cause players to change their behavior. For example, a single defector might receive a higher payoff than a cooperator, but at the same time such behavior lowers the total payoff the group received. A lower level of selfishness ($\zeta <1.0$) now couples the payoff of said defector more tightly to the success of the group. In this study, neither kin relations, or multilevel selection are considered. Similarly, the public goods game as well as other evolutionary games already experience inclusive fitness effects (fit-when-rare for example). The idea is to incentivize an individual to cooperate more by coupling its payoff to that of the group, and study under which circumstances this leads to higher degrees of cooperation. This form of incentivizing cooperation by redistributing rewards obtained at the group level, while not a perfect match, is still most akin to group-level selection. However, since group-level selection requires group level reproduction, we call this mechanism here *inclusiveness* and consequently groups that are inclusive will be called *inclusive groups*.

We think that the hypothetical mechanism of introducing inclusiveness into the PGG could be implemented in social systems as well. Instead of an unconditional basic income, one could offer a basic income linked to the economic success of the social group: *conditional basic income*. While it might be intuitive that sharing the rewards and costs drives cooperation, we need to first confirm the intuition, and secondly there might be a critical point at which the system swings from defection to cooperation. Thus, we will show how different degrees of inclusiveness lead to cooperation, and show what role punishment plays in this context.

## Synergy controls cooperation in the public goods games

We analyze the public goods game following Hintze 2015^6^. Each individual in a group of $k+1$ players, i.e. the focal player and her *k* participants, can either cooperate by making a contribution of 1 unit to a common pool or defect and withhold that contribution. The sum of all contributions in the common pool is multiplied by a synergy factor *r* and and then divided equally among all participants, i.e. cooperators and defectors alike. In the case $1< r < k+1$ a dilemma arises as it is a dominant strategy for the individual to defect whereas mutual cooperation would be most beneficial for all. Punishment^2^, i.e. providing each player with the option to impose a punishment fine $\beta$ on other players who defect, has been studied in order to overcome this dilemma. Punishment comes with a cost $\gamma$ for the punisher and we can observe four types of behavior. Cooperators and defectors that do not punish as well as moralists, i.e. cooperators that punish defectors, and immoralists, i.e. players who punish other defectors while simultaneously defecting themselves. Following^6^ $N_{C}, N_{D}, N_{M}, N_{I}$ denote the numbers of cooperators, defectors, moralists and immoralists and $P_{C}, P_{D}, P_{M}, P_{I}$ the corresponding payoffs.

In our approach we additionally introduce the parameter $\zeta \in [0,1]$ as a level of selfishness such that for $\zeta =0$ only the net earnings are distributed equally among the players and not the initial contributions of 1 unit of cooperating players to the public good whereas for $\zeta =1$ we end up with the original public goods game. Later, when punishment will be considered, the cost and fines will be subtracted from the net earnings before redistribution based on $\zeta$ takes place.

### Critical points without punishment

We first analyze a public goods game with our parameter $\zeta$, but without punishment, i.e. for the punishment fine $\beta$ and the punishment cost $\gamma$ there holds $\beta =\gamma =0$ and hence $N_{M}=N_{I}=0$. We first observe that a group of *n* cooperators generates a group-level payoff, i.e. net earnings, of

$$\begin{aligned} Net = r \cdot n - n \cdot 1 = (r-1) \cdot n \end{aligned}$$

as cooperators contribute 1 unit each and note that as we are working from the point of view of the focal player $n=N_{C}+1$ if the focal player is a cooperator whereas $n=N_{C}$ if the focal player is a defector.

In this setting the payoff of a collaborator is

1 $$\begin{aligned} P_{C} = \zeta (r \frac{N_{C}+1}{k+1} -1) + (1-\zeta ) (r-1) \frac{N_{C}+1}{k+1} \end{aligned}$$

whereas the payoff of a defector is given by

2 $$\begin{aligned} P_{D} = \zeta r \frac{N_{C}}{k+1} + (1-\zeta ) (r-1) \frac{N_{C}}{k+1} \end{aligned}$$

Our new parameter $\zeta \in [0,1]$ allows us to interpolate between two extreme situations: For $\zeta =1$ there is no compensation for contributors and we end up with the well-known payoffs for cooperators and defectors from the classical public goods game whereas for $\zeta = 0$ cooperators are fully compensated for their contributions and only net earnings are redistributed among the group.

Equations (1) and (2) can be simplified to

3 $$\begin{aligned} P_{C} = \frac{N_{C}+1}{k+1} (r+\zeta -1) - \zeta \end{aligned}$$

and

4 $$\begin{aligned} P_{D} = \frac{N_{C}}{k+1} (r+\zeta -1) \end{aligned}$$

In order to find the critical point $r_{C}$ we investigate $P_{C} - P_{D} > 0$ and calculate it to be

5 $$\begin{aligned} r_{C} = \zeta (k+1) - \zeta + 1 = \zeta \cdot k + 1. \end{aligned}$$

Note that for $k=4$ expression (Eq. 5) is very plausible as it means $r_{C} > 1$ for $\zeta =0$ (i.e. only the group-level counts) and $r_{C} > k+1 =5$ for $\zeta =1$ (i.e. original public goods game in which only the individual level counts) whereas we obtain $r_{C} > 3$ for $\zeta =0.5$ (and one can easily check that in this case cooperating is still a dominant strategy even if all $k=4$ neighbours defect).

### Critical points with punishment

We now extend our analyses to the case of punishment following^20^ and^6^. Any defecting individual within a group of $k+1$ players, i.e. each defector and each immoralist, is imposed a punishment fine $\frac{\beta }{k}$ by each punisher in the group. Any punishing individual within a group of $k+1$ players, i.e. each moralist and each immoralist, needs to spend a punishment cost $\frac{\gamma }{k}$ per defecting peer in the group. We note that immoralists do not self-punish in this model.

Following^6^ we abbreviate $\rho _{P} = \frac{N_{M}+N_{I}}{k}$ and interpret it as the density of punishers. In order to find the critical point $r_{C}$ we again investigate $P_{C} - P_{D} > 0$ and calculate it to be

6 $$\begin{aligned} r_{C} = \zeta (1 - \beta \rho _{P}) (k+1) - \zeta + 1 - (1- \zeta ) (\beta + \gamma ) \rho _{P}. \end{aligned}$$

A detailed derivation of this expression is given in the Suppl. Appendix. Note that this result is very plausible as it means $r_{C} > 1 - 1 \cdot (\beta + \gamma ) \cdot \rho _{P}$ for $\zeta =0$ (i.e. only the group-level counts) and $r_{C} > (k+1) \cdot (1 - \beta \rho _{P})$ for $\zeta =1$ (i.e. reproducing the findings from equation (14) from the paper^6^) whereas we obtain our original expression (Eq. 5) for $\beta = \gamma =0$.

We finally observe that for $\zeta \in [0,1[$ our approach incorporates an implicit punishment cost for the cooperators even in the case $\gamma = 0$ as punishment reduces the group-level payoff, i.e.  the net earnings of the group, for both the cooperators and the defectors.

## Computational evolutionary model

The above described mathematical formalism should translate to a system of evolving agents. However, our mathematical formalism (Eqs. 1–6 ) assumes infinite populations and their individual actions to be equivalent to mean play frequencies. Also, evolution is defined as a process comprised of inheritance, variation, and selection. In order to replicate this process accurately, we can not disregard the effect of stochastic and discrete mutations, as we would otherwise not model evolutionary but only population dynamics^21^. Therefore, we confirm our above findings by using a computational agent based evolutionary model. A population of agents plays the above described public goods games and their performances define their reproductive success. The actions of each agent are encoded by genes, specifically by a pair of probabilities. These probabilities define each agent’s likelihood to cooperate $p_{C}$ and to punish $p_{P}$. When agents are selected to transmit offspring into the next generation, their genes (probabilities) can experience mutations. This model is identical to the one used in Hintze 2015^6^ except for the payoff function. Group level reproduction^19^ is not implemented directly, as it would not allow the introduction of the selfishness parameter $\zeta$. A group under group-level selection is either replicated as a whole or not. Instead, payoffs are now distributed among the members of the group depending on the degree of selfishness $\zeta$. In the case of $\zeta =1$ each player’s payoff is independent of the group payoff and thus identical with the classic public goods game. In the case of $\zeta =0$ the reward of the individual is the average payoff of the group (as defined before). Under this condition the group members receive the same payoff and thus have the same reproductive success, even though reproduction is executed on an individual level. We also know that the public goods game is dependent on the synergy factor *r* as well as the punishment fine $\beta$ and the punishment cost $\gamma$. This technically creates a four dimensional parameter space with the axes $\zeta$ (in [0, 1]), *r*, $\beta$, and $\gamma$ (all three in $[0,\infty [$).

### Without punishment

**Figure 1:**
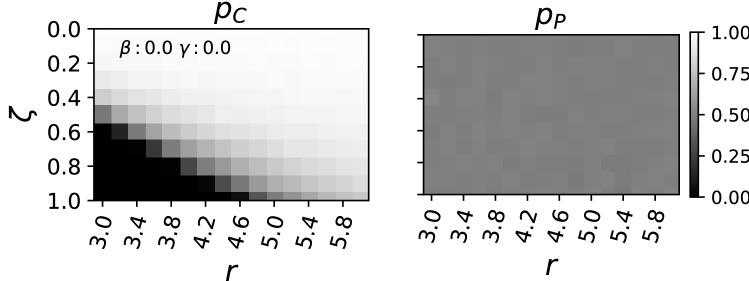
Phase diagram for evolved strategies under varying conditions. In order to model the absence of punishment $\beta$ and $\gamma$ were set to 0.0 while the synergy factor *r* (x-axis) and the degree to which payoffs were distributed individually $\zeta$ (y-axis) were varied. On the left the probability to cooperate at or after the point of convergence is shown, on the right the probability to punish. The color bar on the right shows that probabilities of 0.0 are black, and probabilities of 1.0 are displayed in white. For each square in the phase diagram 100 replicate evolutionary runs over 100.000 generation were performed.

To model a game played without punishment we set $\beta =0$ and $\gamma =0$, and explore only the parameter space for the synergy factor *r* from 3 to 6 (in increments of 0.2) as well as the level of individual payoff $\zeta$ from 0 to 1 (in increments of 0.1). For each combination of factors, we ran 100 independent replicate experiments for 100.000 generations. After the line of descent^22^ was reconstructed, the final 100 generations from all replicate runs were averaged to determine the point of convergence. We find the predictions about the critical points without punishment confirmed (see Fig. 1). For $\zeta =1.0$ we find the critical point for strategies to cooperate at $r=5$, i.e. $k+1=5$. Below that we find strict defection and cooperation to start above that critical point. With an increase of inclusiveness ($\zeta <1$) we find the critical point to move to a lower *r*. As such, cooperation has it easier to evolve the more inclusive groups become. The gene for punishment, as it is neither costly nor rewarding ($\beta =0$ and $\gamma =0$), is drifting, indicated by the genes average value to converge on 0.5.

### With punishment

To confirm the effects of punishment and inclusiveness in the public goods game, six different combinations of cost and fine were tested. Again 100 replicate evolutionary experiments per parameter combination were run and analyzed as before.

**Figure 2:**
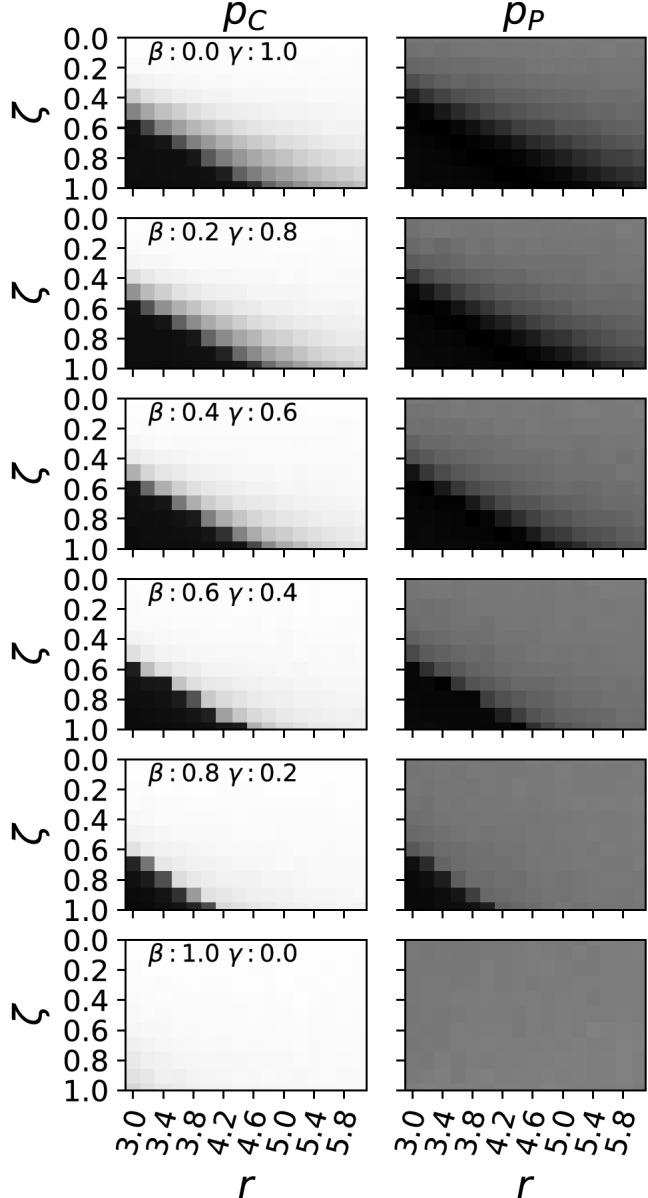
Phase diagrams for evolved strategies under varying conditions for punishment. From top to bottom: The punishment fine $\beta$ was increased over six experiments from 0.0 to 1.0, while at the same time the cost of punishment $\gamma$ was reduced from 1.0 to 0.0. The left column shows the probabilities to cooperate after evolution converged, on the right the same for the probability to punish. Everything else is identical to Fig. 1.

As predicted by the mathematical model, we find the critical point at which cooperation starts to emerge to be dependent on the degree of inclusiveness $\zeta$ as well as on punishment, i.e. the punishment fine $\beta$ and the punishment cost $\gamma$ (see Fig. 2). With an increase of the punishment efficiency, controlled by an increase of the fine $\beta$ and a decrease of the cost for that fine $\gamma$, we find less synergy *r* to be necessary for cooperation to evolve. Similarly, we also observe that evolved strategies do not punish when they also do not cooperate. Consequently, when they do cooperate, the punishment gene starts to drift ($p_{p}=0.5$) as observed before^6^. When all strategies become cooperators and no one punishes, punishment does not happen, and thus no cost is applied, explaining why the punishment gene drifts under those conditions.

In the case where punishment is not costly anymore ($\gamma =0.0$) we find all strategies to become cooperators, and the punishment gene is under drift again.

**Figure 3:**
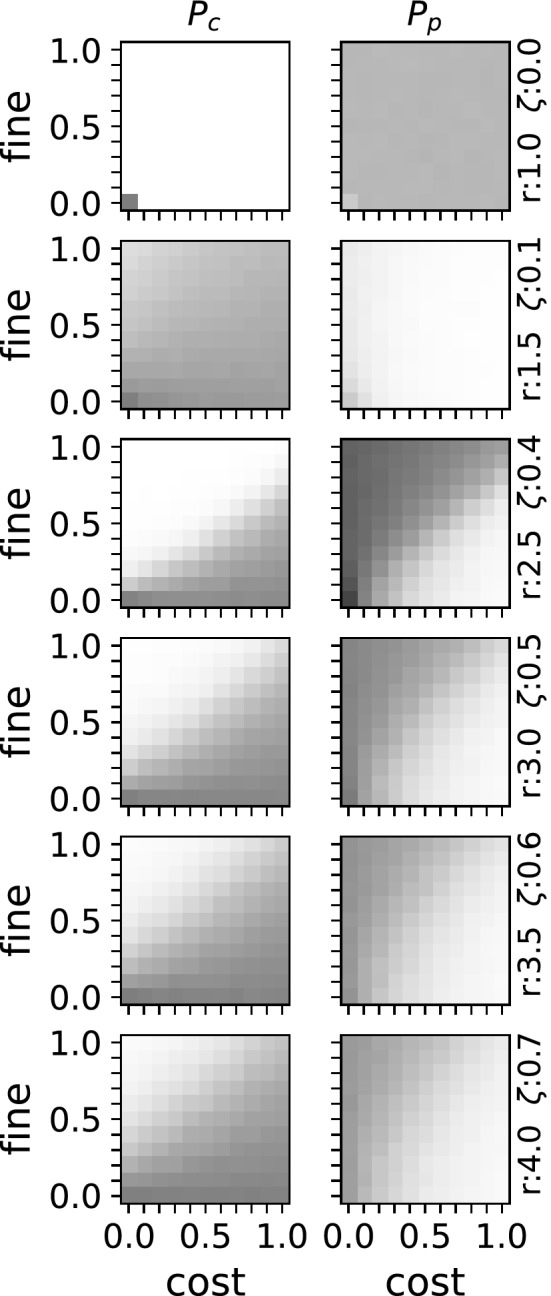
Phase diagrams for evolved strategies exploring the effect of fine $\beta$ and cost $\gamma$ on the critical point. For a selected number of combinations of synergy *r* and selfishness $\zeta$ from top to bottom, see the right side for the specific values, the probability to cooperate is shown on the left ($P_c$) while on the right side the probability to punish $P_p$ is shown. The scale from white (probability of 1.0) to black (probability of 0.0) is used. The data was obtained in the same way as for Fig. 2, the probability to cooperate or punish are the results of the evolutionary process modeled.

**Figure 4:**
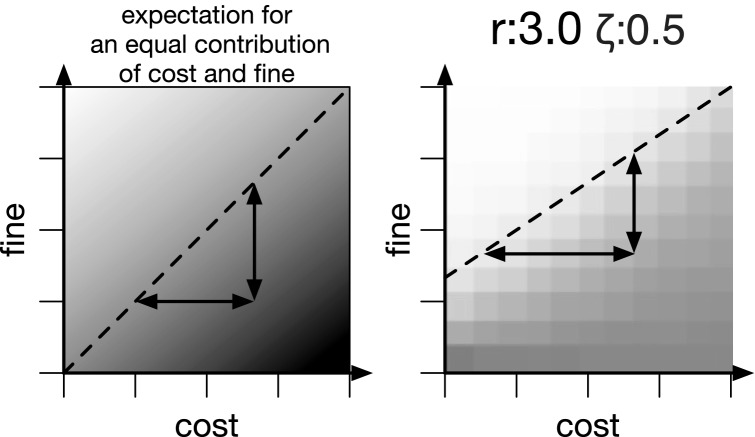
Illustration how fine $\beta$ and cost $\gamma$ affect the critical point differently. When expecting an equal contribution (left panel) changing either cost or fine should be proportional with respect to the change of the critical point (arrows in the left panel are equal), the critical point is illustrated as a dashed line. However, the model results (and the predictions from Eq. 6) suggest a different behavior (right panel for $r=3.0$ and $\zeta =0.5$): changes in cost $\gamma$ must be much larger than changes to fine $\beta$ in order to achieve the same effect on the critical point—illustrated as a dashed line.

Further inspection of the factors determining the critical point (see Eq. 6) suggest a difference between the impact of cost $\gamma$ and fine $\beta$. Specifically, the critical point should be affected more by the fine than the cost of punishment. Results from the computational model confirm this notion (see Fig. 3). For selected values of synergy *r* and degrees of selfishness $\zeta$ already close to the critical point, varying cost and fine shows that cooperation is evolving at high fines and lost costs, while being absent at low fines and high costs as expected. More importantly, varying the cost changes the critical point less than changing the fine does (for an illustration see Fig. 4). As such, the results from the computational model confirm the predictions from Eq. (6).

## Discussion

We introduced a new way to redistribute the payoff in a group of players participating in the public goods game. The degree to which the resources are distributed depend on the degree of inclusiveness of the group. In the case of purely selfish group members, if the synergy between the players is low, defection becomes the optimal strategy. In the case of fully inclusive groups on the other hand, the total payoff the group receives dictates cooperative behavior. The important question answered here is whether resources can be distributed differently and in such a way that individual actions still affect the payoff of the individual while simultaneously coupling the payoff of the individual to the accomplishments of the group. The redistribution of resources according to the degree of individualism $\zeta$ allows for this to happen. We showed mathematically and by using a computational evolutionary model that this form of redistribution indeed promotes cooperation. The lower the degree of selfishness, the sooner group members start to cooperate.

Costly punishment has been identified as an alternative factor that also promotes cooperation. We found that to be true, and also that costly punishment has a synergistic effect when combined with higher levels of group-level selection. However, we also found that the degree of selfishness seems to be a much better way to promote cooperation. When $\zeta <0.5$ we find cooperation ubiquitously present regardless of costly punishment, something only achieved without inclusiveness when punishment becomes free ($\beta =1.0$ and $\gamma =0.0$).

A similar argument is believed to be an important driver for the economy: people are most motivated when their efforts translate into individual gains. Here we showed, that full cooperation, and thus the remedy to the *tragedy of the commons* can already be achieved at $\zeta <0.5$. At higher levels of individualism, costly punishment can be used to achieve the same. This suggests that high levels of taxes combined with an equally fair redistribution of wealth, for example due to a basic income, foster cooperation without the need for costly punishment. The basic income concept suggested here goes beyond providing the means for life’s basic needs. Instead, the wealth of a society is redistributed in such a way, that the beneficiaries can again drive the economy with their spending. In turn, this could grow the economy leading to greater wealth, which can serve as an incentive to spend the basic income such that it further grows the economy^23^. As such, our basic income concept is much more a conditional basic income, as it is conditional on the success of the whole.

In case a higher degree of individualism is desired, punishment can be used as well to promote cooperation, and thus higher total payoffs. Interestingly, as soon as $\zeta <1$ any punishment fine $\beta$ to be paid by punished defectors leads to an implicit punishment cost for any cooperating individuals, too. One may interpret there is less to be distributed within a society that opts for e.g. a larger police force or longer prison sentences. In terms of modeling public goods games our findings imply that the punishment fine $\beta$ is the more important parameter as compared to the punishment cost $\gamma$ which may be forgone unless these costs are desired to be disproportional to the fine $\beta$.

In conclusion, redistribution of resources in such a way that all group members benefit from the success of the group directly can make the tragedy of the commons obsolete. While there are many ways to facilitate this, a conditional basic income that is coupled to the gross domestic product could be one way, even though many other mechanisms of redistribution can be imagined.

## Supplementary information

Supplementary Information.
