# Supplementary material for: Inclusive groups can avoid the tragedy of the commons
Source: Sci Rep. 2020 Dec 28;10:22392. doi: 10.1038/s41598-020-79731-y (PMC7769990; doi:10.1038/s41598-020-79731-y)
Supplement: Supplementary file 1 — Supplementary Information. [file 41598_2020_79731_MOESM1_ESM.pdf]

Arend Hintze[1,2,*], Jochen Staudacher[3], Katja Gelhar[3], Alexander Pothmann[3], Juliana Rasch[3], and Daniel Wildegger[3]

[1]Dalarna University, Institute for Complex Dynamical Systems and MicroData Analytics, Sweden
[2]BEACON Center for the Study of Evolution in Action, Michigan State University, USA
[3]Kempten University of Applied Sciences, Faculty of Computer Science, Germany
[*]corresponding author: ahz@du.se

Appendix

We give a detailed derivation of expression 6 for the critical point r_C in the presence of punishment. We first repeat the payoffs P_C, P_D, P_M, P_I of cooperators, defectors, moralists and immoralists in the classical public goods game, i.e. the case $\zeta = 0$, see[20] and[6].

$$\begin{aligned} P_C &= r\frac{N_C+N_M+1}{k+1} - 1 \\ P_D &= r\frac{N_C+N_M}{k+1} - \beta\frac{N_M+N_I}{k} \\ P_M &= P_C - \gamma\frac{N_D+N_I}{k} \\ P_I &= P_D - \gamma\frac{N_D+N_I}{k} \end{aligned}$$

Any defecting individual within a group of $k+1$ players reduces the group-level payoff by $\frac{\beta+\gamma}{k}$ per each punishing peer in the group as defectors are fined $\frac{\beta}{k}$ by each punisher who in turn needs to spend a punishment cost $\frac{\gamma}{k}$ per defecting peer in the group. We remind the reader of the abbreviation $\rho_P = \frac{N_M+N_I}{k}$ for the density of punishers as in[6].

The group-level payoff, i.e. the net earnings, of a group of $k+1$ players is thus

$$Net_C = (N_C+N_M+1)(r-1) - (\beta+\gamma)\cdot\rho_P\cdot(N_D+N_I)$$

if the focal player is a cooperator and

$$Net_D = (N_C+N_M)(r-1) - (\beta+\gamma)\cdot\rho_P\cdot(N_D+N_I+1)$$

if the focal player is a defector.

In our general model, i.e. the case $\zeta \in [0,1]$, the payoffs P_C, P_D, P_M, P_I of cooperators, defectors, moralists and immoralists become

$$\begin{aligned} P_C &= \zeta(r\frac{N_C+N_M+1}{k+1} - 1) + (1-\zeta)\frac{(N_C+N_M+1)(r-1) - (\beta+\gamma)\cdot\rho_P\cdot(N_D+N_I)}{k+1} \\ P_D &= \zeta(r\frac{N_C+N_M}{k+1} - \beta\rho_P) + (1-\zeta)\frac{(N_C+N_M)(r-1) - (\beta+\gamma)\cdot\rho_P\cdot(N_D+N_I+1)}{k+1} \\ P_M &= P_C - \zeta\gamma\frac{N_D+N_I}{k} \\ P_I &= P_D - \zeta\gamma\frac{N_D+N_I}{k} \end{aligned}$$

Investigating $P_C - P_D > 0$ as in[6] leads to

$$\begin{aligned} \frac{r+\zeta-1}{k+1} - \zeta + \zeta\beta\rho_P + (1-\zeta)\frac{(\beta+\gamma)\rho_P}{k+1} &> 0 \\ \frac{r+\zeta-1+(1-\zeta)(\beta+\gamma)\rho_P}{k+1} &> \zeta(1-\beta\rho_P) \\ r+\zeta-1+(1-\zeta)(\beta+\gamma)\rho_P &> \zeta(1-\beta\rho_P)(k+1) \\ r &> \zeta(1-\beta\rho_P)(k+1) - \zeta + 1 - (1-\zeta)(\beta+\gamma)\rho_P. \end{aligned}$$

and thus we confirm expression (6) for the critical point r_C in the presence of punishment.
